# Measuring in vitro ATPase Activity with High Sensitivity Using Radiolabeled ATP

**DOI:** 10.21769/BioProtoc.4676

**Published:** 2023-05-20

**Authors:** Sarina Veit, Thomas Günther Pomorski

**Affiliations:** 1 Department of Molecular Biochemistry, Faculty of Chemistry and Biochemistry, Ruhr University Bochum, Bochum, Germany; 2 Department of Plant and Environmental Sciences, University of Copenhagen, Frederiksberg, Denmark

**Keywords:** ABC transporter, ATPase, ATP hydrolysis, Radioactive assay, ^32^P-ATP, P-type ATPase

## Abstract

ATPase assays are a common tool for the characterization of purified ATPases. Here, we describe a radioactive [γ-^32^P]-ATP-based approach, utilizing complex formation with molybdate for phase separation of the free phosphate from non-hydrolyzed, intact ATP. The high sensitivity of this assay, compared to common assays such as the Malachite green or NADH-coupled assay, enables the examination of proteins with low ATPase activity or low purification yields. This assay can be used on purified proteins for several applications including the identification of substrates, determination of the effect of mutations on ATPase activity, and testing specific ATPase inhibitors. Furthermore, the protocol outlined here can be adapted to measure the activity of reconstituted ATPases.

Graphical overview

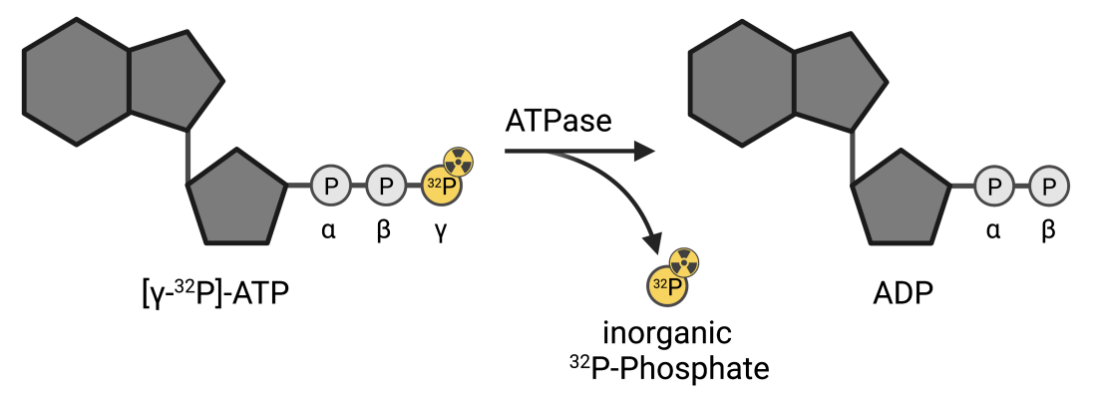

## Background

Through all the kingdoms of life, ATPases are the common enzyme class to catalyze reactions that would otherwise not take place due to high energy barriers. All ATPases use the dephosphorylation of ATP to ADP, resulting in the release of inorganic phosphate. Additionally, the decomposition of ATP provides energy that ATPases use for conformational changes in a variety of ways. Since most ATPases are transmembrane proteins, conformational changes enable the transport of substrates along the membrane and even against the concentration gradient.

Despite intensive research, many details on the mechanism(s) of these ubiquitous enzymes remain to be elucidated. The cellular environment is too complex to investigate one specific protein without the influence of other proteins or molecules. Therefore, most research uses a purification approach to investigate the protein of interest exclusively in the (detergent)-solubilized state or after reconstitution in model membranes, in the case of transmembrane proteins. Since substrate transport is coupled to ATP hydrolysis, one major aspect of the protein characterization is the ATPase activity. Furthermore, it is stimulated by the substrate, pH, ionic strength of the buffer, or in the case of transmembrane proteins, the lipid surroundings.

Many techniques have been used to quantify the in vitro ATPase activity of purified proteins. The most common approach to determine the ATP hydrolysis activity of ATPases is the Malachite green assay, developed in 1966 by Itaya and Ui (Itaya and Ui, 1966). This assay has been improved several times over the last few decades by different groups (Carter and Karl, 1982; Baykov et al., 1988; Geladopoulos et al., 1991; Feng et al., 2011). The method relies on the formation of a green complex when malachite green/molybdate reacts with inorganic phosphate under acidic conditions. The amount of green molybdophosphoric acid complexes can be measured with a spectrophotometer and is directly correlated with the concentration of free inorganic phosphate in the reaction. Another widely used ATP hydrolysis assay is the NADH-coupled assay (Warren et al., 1974). Here, ATP is regenerated by the pyruvate kinase, which simultaneously converts phosphoenolpyruvate to pyruvate. The latter is subsequently converted to lactate by the lactate dehydrogenase and coupled to the oxidation of NADH to NAD^+^. The absorption spectra of both substances at 340 nm vary significantly, allowing live absorbance- or fluorescence-based tracking of NADH consumption, and therefore, indirectly, the coupled ATP hydrolysis (Radnai et al., 2019). Based on the same principle, other fluorescence-based assays use a coupled enzyme system that catalyzes a reaction of a fluorescent substrate with a free phosphate to a product with low fluorescence (Banik and Roy, 1990). Other assays are based on a fluorescent substrate to detect free phosphate in the ATPase reaction that loses its fluorescence by binding to phosphate (Brune et al., 1994). In general, assays utilizing coupled enzyme systems have the disadvantage of being sensitive to the assay conditions, e.g., pH and/or presence of lipids. Furthermore, some ATPases cannot be purified in reasonable quantities, or their ATPase activity is low. Thus, large amounts of purified protein would be needed in the experiment, since neither the Malachite green, the NADH-coupled assay, nor the fluorescence-based assays are sensitive enough with detection limits in the low micromolar or maximum nanomolar range.

Here, we describe an in vitro ATPase assay utilizing radiolabeled [γ-^32^P]-ATP based on previous protocols (Gorbulev et al., 2001), with the ability to detect free phosphate in the femtomolar range. In the assay presented, the active ATPase will liberate radiolabeled gamma-phosphate from [γ-^32^P]-ATP. Excess radiolabeled ATP is subsequently separated from liberated radiolabeled gamma-phosphate by molybdate-phosphate extraction, in which the molybdate-phosphate complex and non-hydrolyzed ATP partition into the organic and aqueous phase, respectively. The radioactive assay provides a direct and sensitive quantification of ATPase activity. In contrast to colorimetric and fluorescent assays, this assay is not disturbed by turbidity, which can be caused, e.g., by detergents and/or lipids present in the samples. The major limitations of radioactive ATPase assays are the hazards of handling radiolabeled isotopes and the unsuitability of this assay format for large-scale high-throughput screening.

## Materials and Reagents

**Biological materials**

The exemplary detergent-solubilized ATPase was a C-terminally GFP-tagged version of the *Cryptococcus neoformans* P4-ATPase Apt1p, in complex with its β-subunit Cdc50p, C-terminally FLAG-tagged (Cdc50p-Flag). The membrane transporter complex was heterologously expressed from a pESC-URA plasmid in the *Saccharomyces cerevisiae* strain *dnf1Δdnf2Δdrs2Δ* (ZHY709, Hua et al., 2002) under the control of a galactose inducible bidirectional promoter. Apt1p-GFP/Cdc50p-Flag was purified via anti-FLAG affinity chromatography, resulting in 1.026 mg/L protein in assay buffer [20 mM HEPES-NaOH, pH 7.5, 20% (w/v) glycerol, 150 mM NaCl] supplemented with 0.04% (w/v) n-Dodecyl-β-D-maltoside (DDM), stored at -80 °C (Stanchev et al., 2021).

*Note: The protocol can also be applied to other ATPases, either purified or reconstituted (Gorbulev et al., 2001; van der Does et al., 2006; Marek et al., 2011; Laub et al., 2017; Theorin et al., 2019). Whether the ATPase activity of the protein remains during storage needs to be tested.*

**Materials**

1. Polypropylene reaction tubes: 1.5, 2, and 15 mL (e.g., Sarstedt, catalog numbers: 72.690.001, 72.691, 62.554.502)
2. 15 mL glass tubes with screwable lid (e.g., Oehmen, catalog numbers: 7920120, 6702588)
*Note: To avoid phosphate contamination, glassware should be washed in phosphate-free detergent prior to use.*
3. Filter tips: 10, 200, and 1,000 μL (e.g., Starlab, catalog numbers: S1120-3810, S1120-8810, S1126-7810)
4. 5 mL pipette tips (e.g., Gilson, catalog number: F161571)
5. 4 mL scintillation vials (PerkinElmer, catalog number: 1200-421)
6. Glass beakers (100 mL)
7. Glass flasks (500 mL)
8. Measuring flasks (100 mL)
9. Magnetic stir bars
10. Safety goggles
11. Gloves

**Chemicals**

1. N-Dodecyl β-maltoside (DDM) (Glycon, catalog number: D97002)
2. 4-(2-hydroxyethyl)-1-piperazineethanesulfonic acid (HEPES) (Roth, catalog number: 6763.3)
3. Sodium chloride (NaCl) (Fisher Scientific, catalog number: 10284640)
4. Glycerol, >99% (VWR, catalog number: 24388.295)
5. Sodium hydroxide (NaOH) (Signa-Aldrich, catalog number: 30620)
6. Magnesium chloride hexahydrate (MgCl_2_) (Sigma-Aldrich, catalog number: M2670)
7. Isobutanol, 99% (Alfa Aesar, catalog number: B23091)
8. Cyclohexane, ≥99.5% (VWR, catalog number: 23224.293)
9. Acetone, ≥99.5% (Sigma-Aldrich, catalog number: 904082)
10. Adenosine-5’-triphosphate disodium salt hydrate (non-radioactive ATP) (Sigma, catalog number: A3377)
11. Ammonium heptamolybdate tetrahydrate, ≥99% (Roth, catalog number: 3666.1)
12. Scintillation fluid for hydrophilic and lipophilic samples (Roth, catalog number: 0016.3)
13. Hydrochloric acid (HCl) (VWR, catalog number: 20252.290)
14. 85% (w/w) orthophosphoric acid (H_3_PO_4_) (VWR, catalog number: 20624.295)
15. Potassium hydroxide (KOH) (Fisher Scientific, catalog number: P564060)
16. [γ-^32^P]-ATP 3,000 Ci/mmol, 5 mCi/mL, 250 μCi (PerkinElmer, catalog number: BLU502H). Store at 4 °C, half-life is 14.29 days
*Note: We recommend using this 'easy Tide' version, which includes a green dye in the liquid to aid pipetting. Furthermore, this ATP can be stored at 4 °C to avoid freeze-thaw cycles. [γ-^33^P]-ATP can also be used.*
17. Sodium orthovanadate (Sigma, catalog number: 450243)
18. Assay buffer (100 mL) (see Recipes)
19. 20% (w/v) DDM stock (5 mL) (see Recipes)
20. 1 M HCl (100 mL) (see Recipes)
21. Reagent A (15 mL) (see Recipes)
22. Reagent B (33.3 mL) (see Recipes)
23. 20 mM H_3_PO_4 _(5 mL) (see Recipes)
24. 100 mM ATP (10 mL) (see Recipes)
25. 500 mM MgCl_2_ (10 mL) (see Recipes)
26. 700 mM orthovanadate (35 mL) (see Recipes)

## Equipment

1. Analytical balance (Sartorius Entris-i II, 220 g/0.1 mg, Buch Holm, catalog number: 4669128)
2. Eppendorf Research^®^ plus pipettes P20, P200, P1000, P5000 (e.g., Eppendorf, catalog numbers: 3123000039, 3123000055, 3123000063, 3123000071)
3. Thermomixer (e.g., Eppendorf, catalog number: 5382000015)
4. β-radiation protection shield (e.g., Thermo Fisher Scientific, catalog number: 6700-1812)
5. Beta Counter (e.g., Berthold, catalog number: LB 1210B)
6. Vortex mixer (e.g., Vortex Genie 2, Scientific Industries Inc., catalog number: SI-0236)
7. Scintillation counter (e.g., MicroBeta2, PerkinElmer, catalog number: 2450-0020) with sample cassette for 4 mL scintillation vials (PerkinElmer, catalog number: 1450-117)
8. pH meter (e.g., SevenCompact S220, Mettler Toledo, catalog number: 30019028)
9. Magnetic stir platform with heating option (e.g., RCT basic, IKA, catalog number: 0025005927)
10. Fume hood

## Software

1. MicroBeta2 Windows Workstation Version 2.3.0.12
2. Microsoft Excel

## Procedure

**Figure 1.**
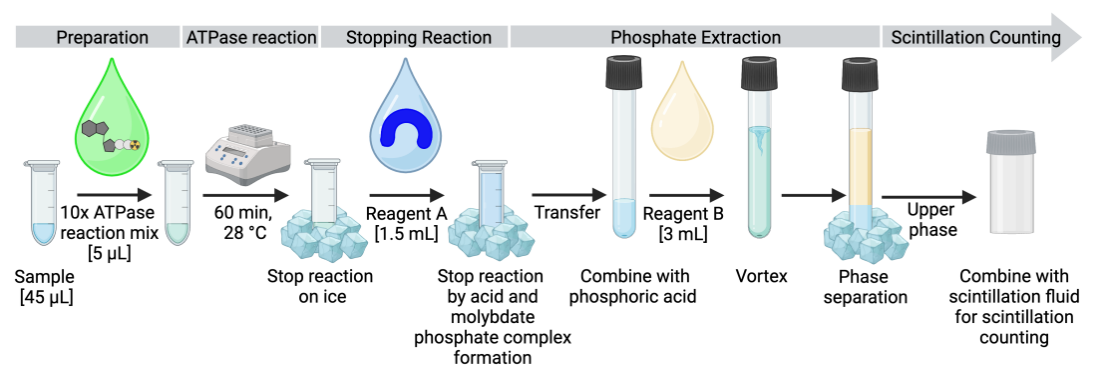
Experimental design and workflow for measuring ATPase activity. After preparing the samples, the ATPase mix is added, and the reaction is started by transferring the samples to the heating block. The reaction is terminated by placing the samples on ice and adding reagent A, which contains molybdate that forms a complex with free inorganic phosphate. The solution is transferred to a glass tube containing orthophosphoric acid, and reagent B is added for phosphate extraction by phase separation. After separation, 500 μL of the upper phase is added to the scintillation fluid in the scintillation vial and taken for measurement.

This assay is performed by mixing the purified or reconstituted ATPase, 5 μCi [γ-^32^P]-ATP, and 1 mM non-radioactive ATP in a reaction buffer that contains Mg^2+^. Control reactions include a sample without protein addition and an incubation of the ATPase in the presence of 1 mM orthovanadate, a classical inhibitor of P-type ATPases. In addition, a catalytically inactive mutant of the protein can serve as a negative control for the reaction to evaluate the presence of potentially contaminating ATPases present in protein preparations. The amount of non-radioactive ATP used in each reaction has to be in excess, so that it is not rate-limiting. Typically, approximately 1 mM of non-radioactive ATP is used per reaction. However, the quantity of protein, the ATP concentration, and the incubation time for the ATP hydrolysis reaction should initially be tested. For an overview, the workflow is visualized in Figure 1. The ATPase reaction is terminated by placing the samples on ice and acid addition. Subsequently, reaction components are separated by molybdate-phosphate extraction and the amount of liberated inorganic phosphate is determined via scintillation counting. Comparison to a buffer sample without protein is used for background correction. A calibration reference sample with a known volume from the [γ-^32^P]-ATP stock allows calibration of the measured scintillation signal to [γ-^32^P] in the sample, and thus a quantification of the hydrolyzed ATP.

- **Sample preparation**
  We recommend preparing each sample as duplicates or triplicates.
  1. Prepare test samples with a final volume of 45 μL in separate 2 mL reaction tubes on ice. An exemplary pipetting scheme is shown in Table 1. Typically, 40–250 ng of purified ATPase is used per reaction. If the specific ATPase activity range of the protein is unknown, several dilutions should be tested. Use the assay buffer to make a total of 45 μL volume.
*Note: It is recommended to store purified proteins at -80 °C in small aliquots to avoid multiple freeze-thaws. Thawing has to be done slowly on ice before an experiment is performed.*
    **Table 1.** Exemplary pipetting scheme for samples and buffer in duplicates and calibration reference in triplicates. 10*×* ATPase reaction mix is added at a later step (see Section C).

Sample	Repl.	Protein (μL)	Inhibitor (μL)	Buffer (μL)	10*×* ATPase reaction mix (μL)
Buffer	I	-	-	45	5
	II	-	-	45	5
Sample	I	39	-	6	5
	II	39	-	6	5
Sample + inhibitor	I	39	1	5	5
	II	39	1	5	5
Calibration reference	I	-	-	-	0.2 (2 μL of 1:10 dilution)
	II	-	-	-	0.2 (2 μL of 1:10 dilution)
	III	-	-	-	0.2 (2 μL of 1:10 dilution)
  2. Prepare test samples with inhibitor using the same quantity of purified ATPase as before with the addition of inhibitor. Use the assay buffer to make a total of 45 μL volume. Incubate the samples for 20 min at room temperature to allow proper inhibition.
*Note: If sodium orthovanadate is used as an inhibitor, it has to be boiled for 15 min at 95 °C and subsequently stored on ice until use to destroy polymers.*
  3. Prepare a blank by adding 45 μL of assay buffer in a separate 2 mL reaction tube.
  4. Store samples (after incubation if inhibitors are used) on ice until the assay is started.
- **Preparation of the 10*×* ATPase reaction mix**
  1. To take the rate of decay of [γ-^32^P]-ATP into consideration, calculate its activity for the day the assay is conducted. Manufacturers specify a calibration or reference date that corresponds to the indicated activity of a radiolabeled reagent. This date can be used to determine the residual activity of the radioactive isotope on the day the assay is conducted using the following equation:

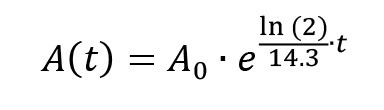

    A(t) = activity at time point *t*
    A_0_ = initial activity (at *t=0)*
    t = time point (days after *t=0*)
    Furthermore, the reference number of the [γ-^32^P]-ATP (BLU502H) can be used to obtain both the concentration in mCi/mL and the specific activity in Ci/mmol of the stock solution via the PerkinElmer Homepage:
https://www.perkinelmer.com/tools/calculatorrad#/product/BLU502H
*Note: The recommended [γ-^32^P]-ATP (BLU502H) contains 50 μL only. The earlier the experiment is performed after the calibration date, the more samples can be tested.*
  2. Calculate the volumes needed for each component for the preparation of the 10*×* ATPase reaction mix (1 mM ATP, 5 mM MgCl_2_, 0.4 μCi/μL [γ-^32^P]-ATP in assay buffer) as displayed in Table 2. For each sample, 5 μL of mix is needed and an excess of one sample should be prepared for the preparation of calibration reference samples. Based on the calculation, first prepare the 10*×* ATPase reaction mix without [γ-^32^P]-ATP.
    **Table 2.** Exemplary pipette scheme for the 10*×* ATPase reaction mix for one and six samples. For every sample, 5 μL of the mix with a total of 2 μCi [γ-^32^P]-ATP is needed. The amount of [γ-^32^P]-ATP has to be calculated on the basis of the half-life and calibration date (exemplified here with a concentration on the date of the experiment of 5 μCi/μL). The total volume of the reaction mix should always be adjusted to 5 μL per sample using assay buffer.

10*×* ATPase reaction mix	per sample (μL)	For 6+1 samples (μL)	Final conc.
ATP (100 mM)	0.5	3.5	1 mM
MgCl_2_ (500 mM)	0.5	3.5	5 mM
Assay buffer	3.6	25.2	-
[γ-^32^P]-ATP	0.4	2.8	0.4 μCi/μL
Total volume	5	35	-
*From this step onwards, radioactive material handling precautions have to be taken. These include the use of appropriate shielding materials such as Perspex shielding (3/8 inches thick), behind which all work should be done, and Perspex Eppendorf tube holders. Surfaces should be routinely monitored by Geiger counters, and ring dosimeters can be used to monitor personal exposure. Radioactive isotopes should be used only by authorized personnel in designated places following the institution’s regulations. Requisition and storage of radioactive material, solid and liquid radioactive waste disposal, and spill decontamination should be carried out according to the institution’s regulations.*
  3. Finalize the 10*×* ATPase reaction mix by addition of the appropriate volume of [γ-^32^P]-ATP from the solution stock, as calculated in B.2.
- **ATPase assay**
  1. Add 5 μL of 10*×* ATPase reaction mix supplemented with [γ-^32^P]-ATP to each sample on ice.
*Note: The excess 10× ATPase reaction mix is later used for the preparation of reference samples in section D.*
  2. Start the ATPase reaction by transferring the samples to a thermomixer at 28 °C under gentle shaking (do not exceed 700 rpm).
*Note: The optimal temperature will depend on the ATPase and should initially be tested.*
  3. Incubate for 60 min.
*Note: Since the assay is based on end-point determination, it is important that the reaction is not limited by the amount of ATP available. To prevent a complete ATP consumption and thereby underestimation of sample activity, several ATPase dilutions should be tested.*
  4. Stop the reaction by placing the samples on ice and subsequently adding 1.5 mL of Reagent A (Recipe 4).
*Note: All ATPases should be inactivated and the reaction stopped completely. However, to prevent autohydrolysis of [γ-^32^P]-ATP, the samples should be processed directly by extraction of phosphate.*
- **Molybdate-phosphate extraction**
*Note: Molybdate-phosphate extraction is needed to separate inorganic ^32^P-phosphate from non-hydrolyzed ^32^P-ATP, as illustrated in Figure 2.*
  **Figure 2.**
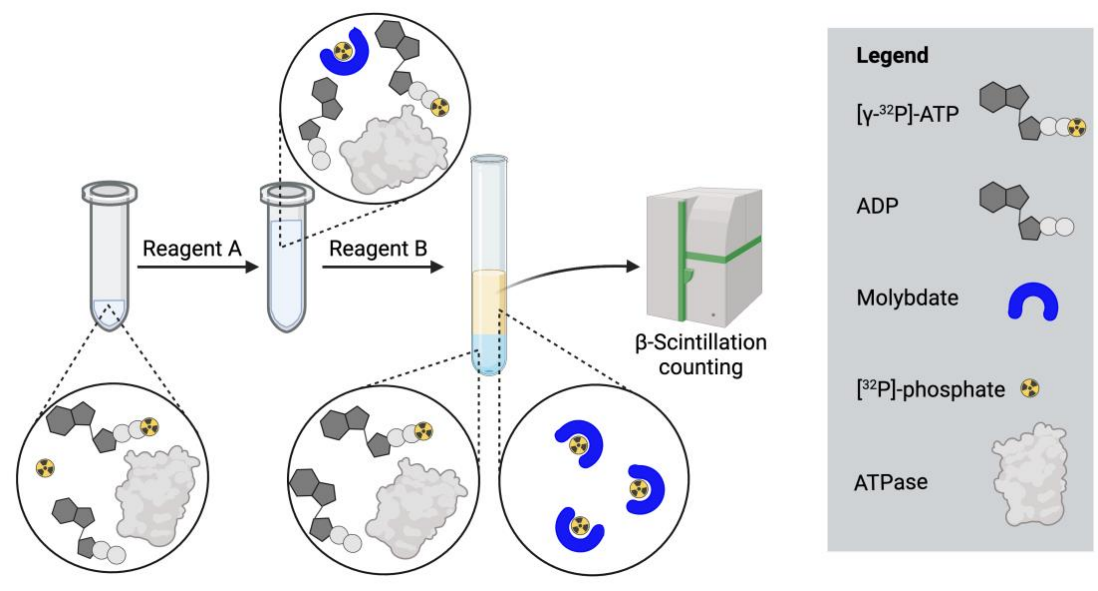
Illustration of the molybdate-phosphate extraction procedure. After the ATPase reaction, free ^32^P-phosphate and non-hydrolyzed γ-^32^P-ATP are present in the same solution. Reagent A, containing ammonium molybdate, is added and forms a complex with the inorganic phosphate. Addition of reagent B results in phase separation in the lower aqueous phase and the upper organic phase. The molybdate-phosphate complexes localize to the upper organic phase, while the non-hydrolyzed ATP remains in the aqueous phase. A sample of the organic phase is taken for scintillation counting, to determine the amount of inorganic phosphate formed.
  1. Transfer each sample to separate 15 mL glass tubes containing 15 μL of 20 mM H_3_PO_4 _(Recipe 6).
*Note: The phosphate of H_3_PO_4 _serves as a carrier for the inorganic phosphate of the ATPase reaction in the next step.*
    Add 3 mL of Reagent B to all samples, fasten the lid, and vortex for 30 s.
*Note: Reagent B is volatile. Work under the fume hood.*
  2. Incubate on ice for 10 min to allow phase separation.
  3. Add 2 mL of scintillation fluid to 4 mL scintillation vials; separate tubes are needed for every sample and three calibration references.
*Note: The scintillation counter used here measures through the lid. Therefore, the tubes should be labeled on the sides of the vial, not on the lid. Clarify how your device is measuring and change the labeling position accordingly.*
  4. After phase separation, take 500 μL of yellow, upper, organic phase containing the radioactive orthophosphate-molybdate complex and add it on top of the scintillation fluid in scintillation vials.
*Note: The organic phase has low surface tension and tends to drop out of the pipette.*
*Note: Do not pipette up and down in the vial, as scintillation fluid gets stuck in the tip.*
  5. To allow quantification of the scintillation measurement, prepare a 1:10 dilution of 10*×* ATP reaction mix (2 μL + 18 μL H_2_O) and add 2 μL of 1:10 dilution to the scintillation tube with scintillation fluid. Make triplicates of this calibration reference.
- **Scintillation counting using MicroBeta2**
  1. Tighten the lids of the scintillation vials properly.
  2. Place vials in the measuring cassette, invert it several times for mixing, and place it in the scintillation counter with the stop cassette below.
  3. Start the MicroBeta2 Windows Workstation program.
  4. Create a protocol for ^32^P measurement, as shown in Figure 3.
    - Set Label 1 to ^32^P and Label 2 to None.
    - Set Plate/Filter to 4 mL plate 6 × 4.
    - Set Assay Type to Normal.
    - Set counting time to 1 min.
    - Set Detector Normalization and Background correction to None.
    - Set counting precision to 0.2 2 sigma%.
    - Set cycling parameter to 1 time per plate.
    **Figure 3.**
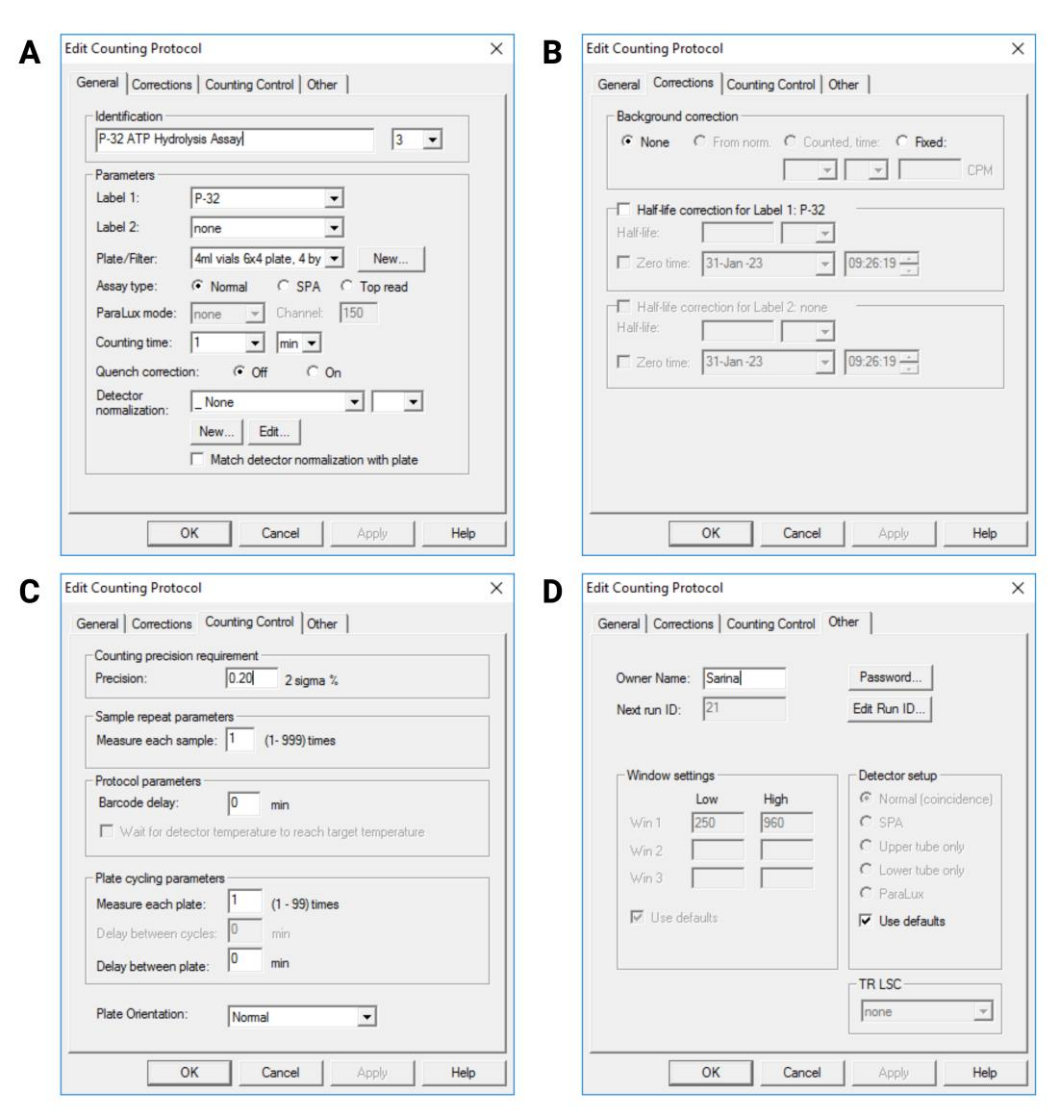
User interface of MicroBeta2 Windows Workstation program showing the protocol settings. A: General settings; B: Correction settings; C: Counting Control settings; D: Other settings.
  5. Change the plate setup to only measure the slots that include a vial.
  6. Start the measurement.
  7. Extract the cpm values per sample as .txt files.

## Data analysis

1. Extract the signal intensities from the .txt file and load it into Microsoft Excel for analysis.
2. Data analysis:
  - First, the amount of hot ATP in the reference sample must be calculated based on its specific activity on the day of the experiment and the volume taken from the 10*×* ATPase reaction mix. Correlating the amount of hot ATP in the calibration reference sample with its measured cpm value allows calibration of measured cpm values. Exemplary sample values for calculation are listed in Table 3.
  - Next, the sample signal can be background corrected by the buffer sample signal, and the amount of hydrolyzed hot ATP can be quantified.
  - After correction for the total upper phase volume and extrapolation for hydrolyzed non-radiolabeled ATP, the total amount of hydrolyzed ATP can be specified per minute and per milligram of protein to yield the specific ATPase activity.
    **Table 3.** Exemplary data from scintillation measurement. Calibration reference, buffer and sample are prepared as triplicates and duplicates, respectively. Each sample included 40 ng of protein.

Sample	Sample signal [cpm]
Calibration reference I	221,331
Calibration reference II	218,048
Calibration reference III	221,200
Buffer I	8,874
Buffer II	8,444
Sample I	50,037
Sample II	53,292
  - Reference calculation for quantification:
    For the reference calculation, 0.2 μL was taken from the 10*×* ATPase reaction mix with a concentration of 0.4 μCi/μL [γ-^32^P]-ATP, resulting in 0.08 μCi [γ-^32^P]-ATP.
$$\frac{activity\;in\;reference\;[Ci]}{specific\;activity\;[\frac{Ci}{mmol}]}=hot\;ATP\;in\;reference\;sample\;[mmol]\;$$
$$\frac{0.08\times 10^{-6}Ci\;}{3,000\frac{Ci}{mmol}}=2.667\times 10^{-11}\;mmol\;$$
    In the exemplary experiment, these 2.667 × 10^-11^ mmol of the reference sample referred to the calibration reference average of 220,193 cpm.
  - Sample analysis:
    i. The counts for duplicates of each sample are averaged, and the background correction is applied for the averaged background signal.
$$av.\;sample\;signal\;\left[cpm\right]-av.\;buffer\;signal\;\left[cpm\right]=corr.\;sample\;signal\;\left[cpm\right]$$
$$\frac{50,037\;cpm+53,292\;cpm}{2}-\frac{8,874\;cpm+8,444\;cpm}{2}=43,006\;cpm$$
*Note: The buffer serves not only as a control for the spurious extraction of intact [γ-^32^P]-ATP in the upper organic phase, but also for the amount of [γ-^32^P]-ATP that is already hydrolyzed in the stock or in the time course of the experiment, as well as phosphate contaminations in the used solutions.*
    ii. The buffer corrected counts for each sample are normalized to millimole of hydrolyzed hot ATP via the reference calculation.
$$\frac{hot\;ATP\;in\;reference\;sample\;\left[mmol\right]}{av.\;reference\;sample\;\left[cpm\right]}\times sample\;\left[cpm\right]=hydrolzyed\;hot\;ATP\;[mmol]$$
$$\frac{2.667\times 10^{-11}\;mmol}{220,193\;cpm}\times 43,006\;cpm=5,208\times 10^{-12}\;mmol$$
    iii. The calculated amount of hydrolyzed hot ATP refers to 500 μL taken from the upper phase for analysis only. Therefore, for the complete upper organic phase of 3 mL, the calculated value has to be multiplied by six, the volume correction factor (${CF}_{Vol}$).
$$hydr.\;hot\;ATP\;\left[mmol\right]\times {CF}_{Vol}=total\;\;hydr.\;hot\;ATP\;\left[mmol\right]\;$$
$$5,208\times 10^{-12\;}mmol\times 6=3,124\times 10^{-11}\;mmol$$
    iv. Since the ratio of [γ-^32^P]-ATP to non-radiolabeled ATP was 1:75,000 (mol:mol), the calculated value has to be further multiplied by 75,000, the ratio correction factor (${CF}_{Ratio})$, to account for all ATP present.
$$total\;hydr.\;hot\;ATP\;\left[mmol\right]\times {CF}_{Ratio}=total\;hydr.\;ATP\;\left[mmol\right]\;$$
$$3,124\times 10^{-11}\;mmol\times 75,000=\;{2,344\times 10}^{-6}\;mmol$$
    v. In the last step, the calculated amount of hydrolyzed ATP has to be normalized for the amount of protein ($m_{protein})$ in the sample and for the reaction time of 60 min, resulting in a final specific ATPase activity in millimole hydrolyzed ATP per milligram of protein per minute.
$$\frac{total\;hydr.\;ATP\;\left[mmol\right]}{reaction\;time\;\left[min\right]}/m_{protein}\;[mg]=spec.\;Activity\;[mmol/min/mg]$$
$$\frac{{2,344\times 10}^{-6}\;mmol}{60\;min\;}/0.04\times 10^{-3}\;mg=976.5\times 10^{-6}\;[mmol/min/mg]$$
    vi. In summary, the specific activity can be calculated based on the background corrected cpm values as follows:
$$\frac{\frac{hot\;ATP\;in\;ref.\;\left[mmol\right]}{\;ref.\;\left[cpm\right]}\times corr.\;signal\;\left[cpm\right]\times {CF}_{Vol}\times {CF}_{Ratio}\;}{Reaction\;time\;[min]}/m_{protein}\;\left[mg\right]=specific\;ATPase\;activity\;[mmol/mg/min]$$
    ref. = reference sample
    CF_Vol_ = correction factor for total upper phase volume
    CF_Ratio_ = correction factor for ratio hot to cold ATP
    m_protein _= amount of protein
    vii. The final specific ATPase activity can be compared between samples with and without an inhibitor. Here, the sample without inhibitor is set to 100% to allow quantification of specific inhibition in percentage (Figure 4).
    **Figure 4.**
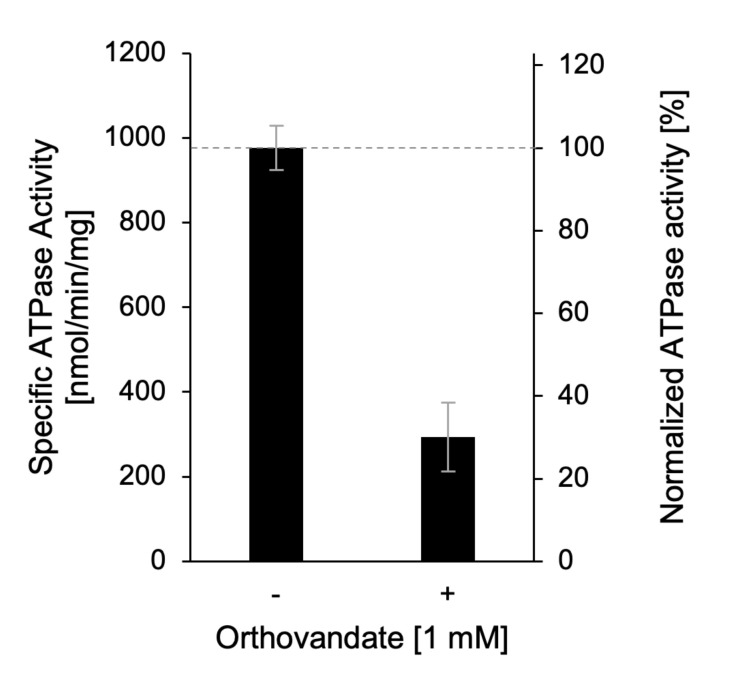
Exemplary result of ATPase assay with detergent-solubilized Apt1p/Cdc50p. ATPase activity of purified Apt1p/Cdc50p assayed in the absence (-) and presence (+) of 1 mM orthovanadate, an inhibitor of P-type ATPases. Adapted from Stanchev et al. (2021).

## Recipes

*Note: Since low quantities of free phosphate are quantified, a phosphate contamination of the used reagents and material should be minimized. A control sample only containing buffer should be used for detection of the background signal and data correction.*

1. **Assay buffer (100 mL)**
  20 mM HEPES (4.766 g), 150 mM NaCl (8.766 g), 20% (w/v) glycerol (20 g), 0.05% (w/v) DDM (250 μL of 20% stock; see Recipe 2), pH 7.4.
  Weigh NaCl, glycerol, and HEPES and fill up to 90 mL with ddH_2_O. Adjust the pH with 1 M NaOH to 7.4. Add DDM and fill up to 100 mL with ddH_2_O.
*Note: For other ATPases, this buffer may be adapted.*
2. **20% (w/v) DDM stock (5 mL)**
  Dissolve 1 g of DDM in 3 mL of ddH_2_O in a 15 mL polypropylene screw-cap tube and incubate with head-over-head rotation until completely dissolved. Let the foam set and fill up to a total of 5 mL with ddH_2_O. Store at 4 °C.
3. **1 M HCl (100 mL)**
  Dilute 8.3 mL of concentrated (37%) HCl to a total volume of 100 mL with ddH_2_O.
4. **Reagent A (15 mL)**
  Dissolve 0.185 g of ammonium heptamolybdate tetrahydrate in a total volume of 15 mL in 1 M HCl.
*Note: Prepare fresh on the day of the experiment. Per sample, 1.5 mL of Reagent A is needed. We recommend preparing a sufficient volume for one extra sample.*
5. **Reagent B (33.3 mL)**
  Mix isobutanol, cyclohexane, acetone, and Reagent A in a glass flask in a ratio of 5:5:1:0.1 (v:v) by circular movements, resulting in 45% (v/v) isobutanol (15 mL), 45% (v/v) cyclohexane (15 mL), 9% (v/v) acetone (3 mL), and 0.1% Reagent A (0.3 mL) for 33.3 mL.
*Note: Substances used are volatile and toxic. Work under the fume hood. Prepare fresh on the day of the experiment. Per sample, 3 mL of Reagent B is needed. We recommend preparing a sufficient volume for one extra sample.*
6. **20 mM H_3_PO_4_ (5 mL)**
  Dilute 6.5 μL of 85% (w/w) orthophosphoric acid to a total of 5 mL in ddH_2_O.
7. **100 mM ATP (10 mL)**
  Dissolve 551 mg of ATP in a total volume of 8 mL in ddH_2_O. Adjust pH to 7.0 with 1 M KOH. Fill up to 10 mL with ddH_2_O. Freeze aliquots of e.g., 200 μL in 1.5 mL polypropylene reaction tubes at -20 °C. Avoid repeated freeze-thawing.
8. **500 mM MgCl_2_ (10 mL)**
  Dissolve 1.017 g of MgCl_2_ hexahydrate in a total volume of 10 mL in ddH_2_O. Store at RT.
9. **700 mM orthovanadate (35 mL)**
  - Add 4.5 g of sodium orthovanadate to 30 mL of ddH_2_O in a glass beaker and stir until completely dissolved.
    Adjust the pH to 10 using 1 M NaOH or 1 M HCl.
*Note: Acidification leads to a color change to yellow.*
  - Boil the solution on a heating plate with continuous stirring until the solution loses its color.
  - Let cool to room temperature and measure pH.
  - Titrate again to pH 10 using 1 M NaOH or 1 M HCl and repeat boiling and titration until pH = 10 is stable at room temperature.
  - Fill up with ddH_2_O to 35 mL.
  - Store aliquots of e.g., 200 μL in 1.5 mL polypropylene reaction tubes at -20 °C.
  - Directly before use, heat the solution for 15 min at 95 °C to break polymeric species (Goodno, 1982) and place it on ice until use.
*Note: Orthovanadate is toxic. Work under the fume hood and wear protective gear.*
